# Picking up the hydrothermal whisper at Ischia Island in the Covid-19 lockdown quiet

**DOI:** 10.1038/s41598-021-88266-9

**Published:** 2021-04-23

**Authors:** Mariarosaria Falanga, Paola Cusano, Enza De Lauro, Simona Petrosino

**Affiliations:** 1 Dipartimento Di Ingegneria Dell’Informazione Ed Elettrica E Matematica Applicata/DIEM, Università Degli Studi Di Salerno, Fisciano, Italy; 2 Istituto Nazionale Di Geofisica E Vulcanologia, Sezione Di Napoli - Osservatorio Vesuviano, Naples, Italy; 3 Ministry of Education, Universities and Research, Rome, Italy

**Keywords:** Seismology, Volcanology

## Abstract

In this paper, we analyse the seismic noise at Ischia Island (Italy) with the objective of detecting the hydrothermal source signals taking advantage of the Covid-19 quiescence due to lockdown (strong reduction of anthropogenic noise). We compare the characteristics of the background noise in pre-, during and post-lockdown in terms of spectral content, energy release (RMS) and statistical moments. The continuous noise is decomposed into two independent signals in the 1−2 Hz and 2−4 Hz frequency bands, becoming sharpened around 1 Hz and 3 Hz respectively in lockdown. We propose a conceptual model according to which a dendritic system of fluid-permeated fractures plays as neighbour closed organ pipes, for which the fundamental mode provides the persistent whisper and the first higher mode is activated in concomitance with energy increases. By assuming reasonable values for the sound speed in low vapor–liquid mass fraction for a two-phase fluid and considering temperatures and pressures of the shallow aquifer fed by sea, meteoric and deep hydrothermal fluids, we estimate pipe lengths in the range 200–300 m. In this scheme, Ischia *organ-like* system can play both continuous whisper and transients, depending on the energy variations sourced by pressure fluctuations in the hydrothermal fluids.

## Introduction

The Covid-19 pandemic has produced huge repercussions on human activities all over the world. Most part of the countries adopted drastic emergency measures to reduce the diffusion of the disease, including the closure of industries, public facilities, services, and infrastructures, as well as travel restrictions, physical distancing, and quarantine. After China, where the coronavirus was first identified in December 2019, Italy was one of the first countries struck by the pandemic. In order to contain the devastating effects of the infection, on March 9, 2020, the Italian government imposed a lockdown consisting in severe restrictions which were gradually eased since May 4, 2020.

Scholars, in any research field, took this unique opportunity to study their topics in situations of reduced noise amplitude, from seismology^1^ to acoustic environment^2^ and atmosphere sciences^3^. From a seismological point of view, the lockdown period represented a suitable circumstance to tackle the study of the seismic noise from a new perspective; recent researches have shown that the adoption of this restrictive measure caused a global reduction of anthropogenic noise of up to 50%^1,4^ and a consequent improvement of the detection thresholds of seismic stations^5,6^. Such a long time period of quiescence offered the chance to detect faint signals normally buried in the background noise and to separate anthropogenic and natural components in an easier way. In other words, lockdown has represented an exceptional environment to detect the signature of low energy source signals and how they can interact with other nearby dominant sources.

The identification of different contributions is particularly relevant in volcanic areas because the noise wavefield could contain signals (e.g. tremor) directly related to the volcanic activity (magmatic and/or hydrothermal)^7^. The characterization of the temporal and spatial patterns of these components through seismological investigations such as spectral and polarization analysis can shed light on the dynamics of the volcanic source^8–11^.

**Figure 1:**
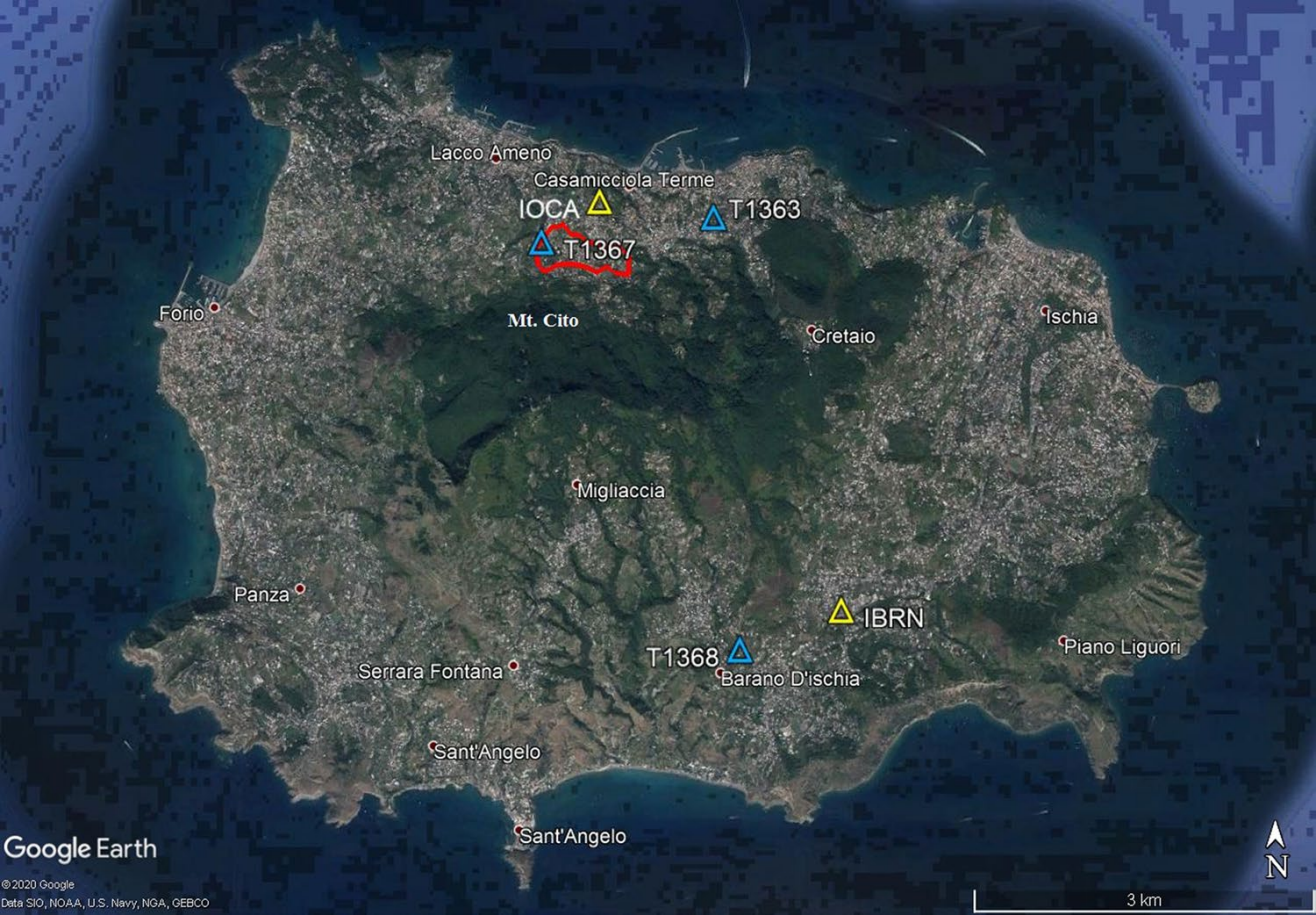
Map of Ischia Island with the stations reported as triangles (Map data 2018 Google retrieved from https://www.google.it/maps/@40.7251646,13.9032732,8719m/data=!3m1!1e3). Permanent stations^22^ are indicated in yellow and temporary ones^23^ in light blue. The red line surrounds the forbidden area (red zone) most struck by the M_D_ 4 earthquake of August 21, 2017.

In this context, we studied the seismic noise acquired at Ischia (Italy), an island belonging to the Phlegrean Volcanic District, Southern Italy^12^, which normally is a travel destination for many Italians and foreign tourists, attracted by its wonderful natural landscape and therapeutic thermal springs. Recent studies carried out at Ischia Island have shown that the seismic noise wavefield at frequencies lower than 5 Hz^13,14^ mainly consists of two persistent independent signals, with dominant frequency peaks in the range 1−2 Hz and 3−4 Hz, respectively. The first one is the most energetic, persistent, and well correlated at the seismic stations located in the Casamicciola area in the northern part of island (see Fig. 1). On the other hand, the amplitude of the second signal shows a modulation on a diurnal scale, indicating that its temporal pattern is somewhat affected by anthropogenic noise. The component at 1−2 Hz has been interpreted by Cusano et al.^13,14^ as the persistent whisper of the shallow circulation of fluids, which are a mixing of sea and meteoric water and thermal fluids of the hydrothermal reservoir^16–19^. In their conceptual scheme, the whispered sound (i.e. the seismic noise) is produced by the interaction of the circulating (hydrothermal) fluids with the solid structure (formed by a network of channels) of the shallow hydrothermal system. This mechanism would generate persistent self-sustained oscillations observed as the 1−2 Hz seismic signal. The second component at 3–4 Hz could represent the higher mode of the hydrothermal source, anyway, modulated by anthropogenic noise. The Covid-19 silence allows us to better clarify the nature of these signals.

Indeed, as elsewhere, the anthropogenic noise at Ischia is a disturbing element that can introduce ambiguities in the analysis of the source components of the wavefield. Therefore, we investigated further the seismic noise detected at Ischia during the lockdown and compared the results with those obtained in the periods of “normal” activity. Ischia experienced a particularly severe lockdown, since the President of the Campania region, to which the island belongs, adopted measures (e.g. stop to all not-essential activities, people forced to stay at home, movements in/across the own town allowed only in few exceptional cases) even more restrictive compared with the national ones, in order to contain the Covid-19 outbreak. When the Italian Prime Minister announced the so-called "Phase 2" (movements at least between municipalities of the same region only for work and health reasons allowed from May 4, 2020; opening of commercial activities from May 18, 2020) and “Phase 3” (free circulation of Italian citizens among regions from June, 2020), the access to Ischia was still totally forbidden, so the island remained completely isolated for two months more after the lockdown ended.

In the present work, we investigate the seismic noise continuously recorded form January 01, 2020 to June 19, 2020 by five seismic stations deployed on the island. Following the well-established approach used in Cusano et al.^13,14^, we applied spectral (FFT) and Independent Component Analysis (ICA^19,20^), and derived the average energy release of the continuous signal on hourly scales^21^. In addition, we focused on the energy distribution of the seismic noise which was investigated by means of low- and high-order statistical analysis. Then, a conceptual model suitable to interpret our observations is proposed.

## Results

### Spectral analysis

Relevant information can be extracted by performing a spectral analysis during the three periods pre- (from January 1 to March 8, 2020), during (from March 9 to May 17, 2020) and post-lockdown (from May 18 to June 19, 2020) on the dataset acquired by the five seismic stations shown in Fig. 1. In this figure, the red line delineates the area most struck by the M_D_ 4 earthquake^14^ of August 21, 2017, which is deserted and not accessible to people since that date because of unsafe buildings (red zone). The spectral analysis was carried out by estimating the standard power spectral density (PSD), for each component of all the operating stations, on samples of 30 s-long time windows spanning throughout all the dataset.

**Figure 2:**
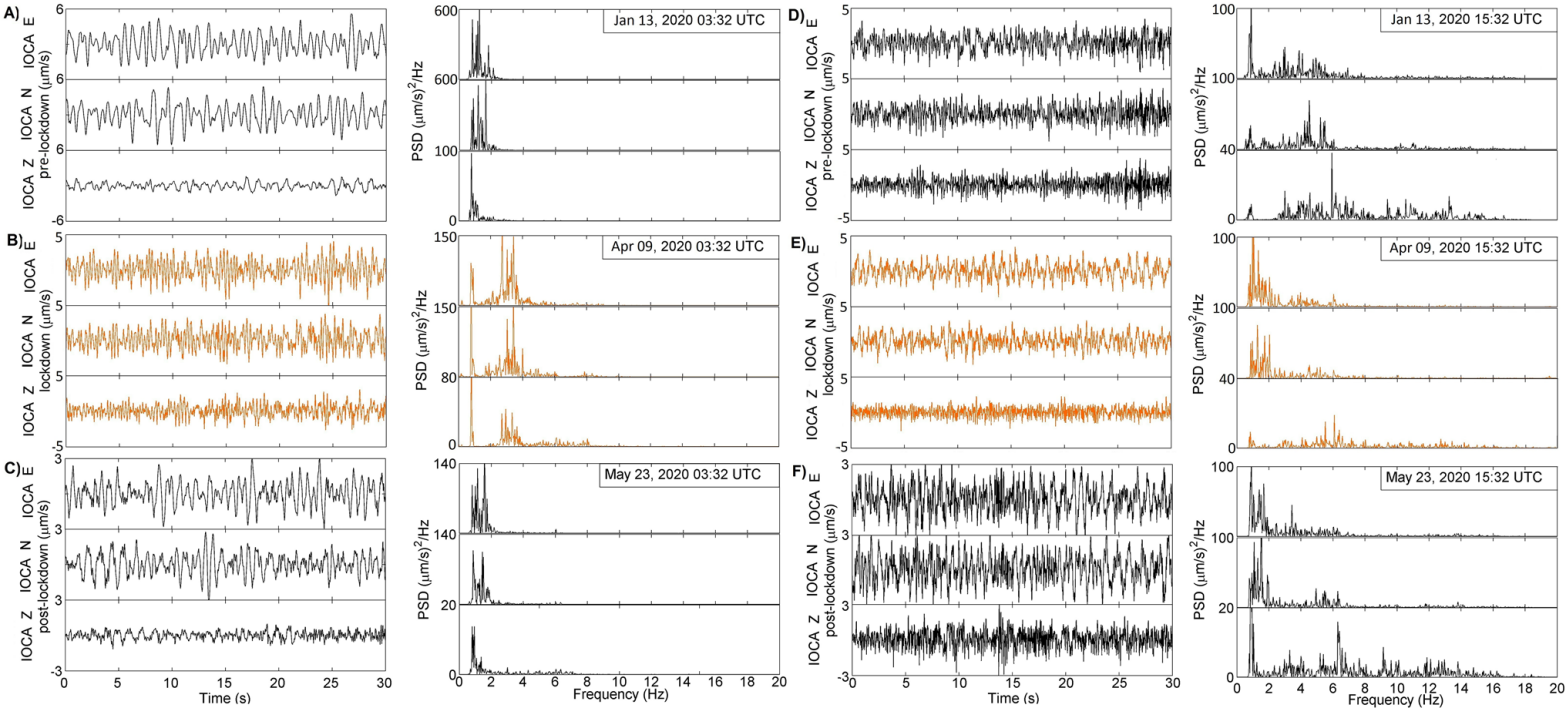
Signals and PSDs acquired at IOCA station during pre- (panel **A**, nighttime, and **D**, daytime), lockdown (panel **B**, nighttime, and **E**, daytime) and post-lockdown (panel **C**, nighttime, and **F**, daytime).

Figure 2 reports an example of signals recorded by the reference station IOCA (Fig. 1) with the relative PSD along the three directions of motion in the period pre-, lockdown and post-lockdown, at the same hours in nighttime (panel A, B and C) and in daytime (panel D, E and F). The spectra evaluated on signals acquired in the post-lockdown (Phase 3) are like those of the pre-lockdown. The spectral content is mainly contained below 10 Hz, with characteristic peaks in the bands 1–2 Hz (b1, hereinafter) and 2–4 Hz (b2, hereinafter). As already observed worldwide^4^, during the lockdown, the amplitude of the anthropogenic noise experiences a reduction (compare panel D, E and F of Fig. 2 as an example) and this allows to evidence other eventual non-anthropogenic signals especially in b2. Following the results of Cusano et al.^13^, the first band contains the signature of a permanent hydrothermal source, whereas not a definitive indication was ascertained on the nature of the second band because affected by the anthropogenic noise. The lockdown silence gives the possibility to better investigate if it is related to the hydrothermal seismic source, in terms of higher harmonics, or strictly caused by the noise.

### RMS amplitude and energy release

**Figure 3:**
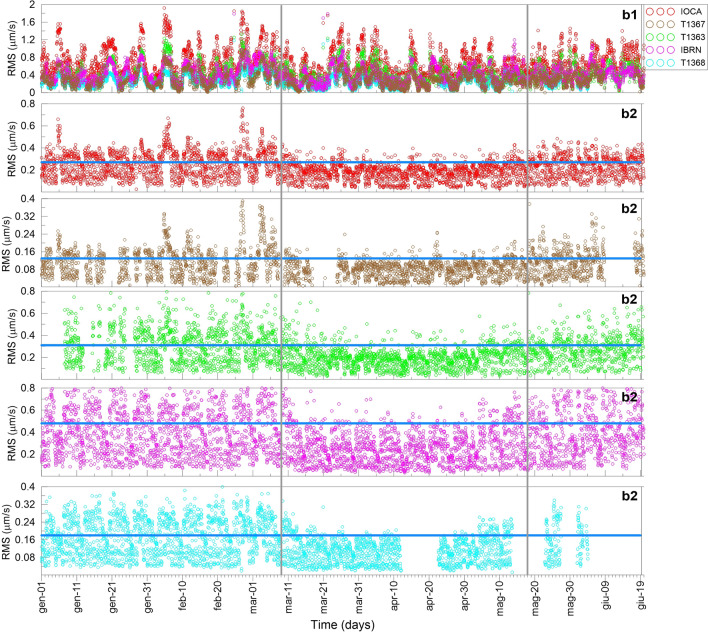
Time evolution of the RMS averaged over 1-h-long samples of the seismic noise recorded in the time interval January-June 2020. The upper panel shows the RMS in b1 for all the seismic stations. The RMS in b2 for each station is shown in the five remaining panels. Missing values are due to sensor breakdowns. The grey vertical lines indicate the beginning (March 9, 2020) of the lockdown and the end of the restriction measures (May 18, 2020); the blue horizontal line represents the 90^th^ percentile of the RMS distribution in the lockdown period.

The analysis of the Root Mean Square (RMS) over a continuous signal (from days to months) can highlight eventual variations of the energy release. Indeed, the squared amplitude of a seismic signal is proportional to its energy^13,14,21,24,25^. We computed the RMS of 1 h-long samples continuously recorded during the first six months of 2020 by 5 stations, thus covering pre-lockdown, lockdown and Phase 3. On the basis of the recent outcomes of Cusano et al.^13^ and considering the results of the previous spectral analysis, data were filtered in the 1–2 and 2–4 Hz frequency ranges, thus evaluating the RMS energy release separately in the two bands. RMS values were, then, averaged over the three directions of motion to point to a coarse-grained variable^25^. In Fig. 3, the obtained RMS values in b1 and b2 are plotted for all the stations.

The upper panel indicates that the RMS in b1 is almost stationary at all the stations all over the investigated time interval, meaning that it does not suffer any anthropogenic noise contribution occurred during the three periods. The other five panels, instead, show the time pattern of the RMS in b2 separately at all the stations. The horizontal blue line indicates the 90^th^ percentile level in the lockdown phase, which roughly corresponds to the mean value of the RMS pre/post-lockdown. As it can be seen, an evident drop of the amplitude occurs during the lockdown, as expected in the case of reduction of noise due to the human activity (such as tourists, workers). Nevertheless, a contribution to the energy in b2 is not negligible and, especially in the North (i.e., at IOCA, T1363, T1367 stations), it is surely poorly affected by the anthropogenic noise. Indeed, the strong periodicities related to both the day/night and weekly activity are much less marked except for the stations located in the South, where a dependence on the human activity cycle, i.e. 24h and 7 days, is still evident. Moreover, during the lockdown, some outliers, above the 90^th^ percentile threshold, emerge from the overall pattern and they have to be investigated in a more detail*.*

### Statistical moments evaluation: skewness and kurtosis

The characteristics of continuous seismic signals can be better highlighted by analyzing the third- and fourth-order statistical moments, skewness *s* and kurtosis *k*^19,26^. In particular, the value of the kurtosis is likely to be the most effective property for identifying deviations from the normal (Gaussian) distribution^27,28^, for which *k* is equal to 3. We calculated skewness and kurtosis of the RMS distribution in the two frequency bands during the pre- and lockdown phases.

**Figure 4:**
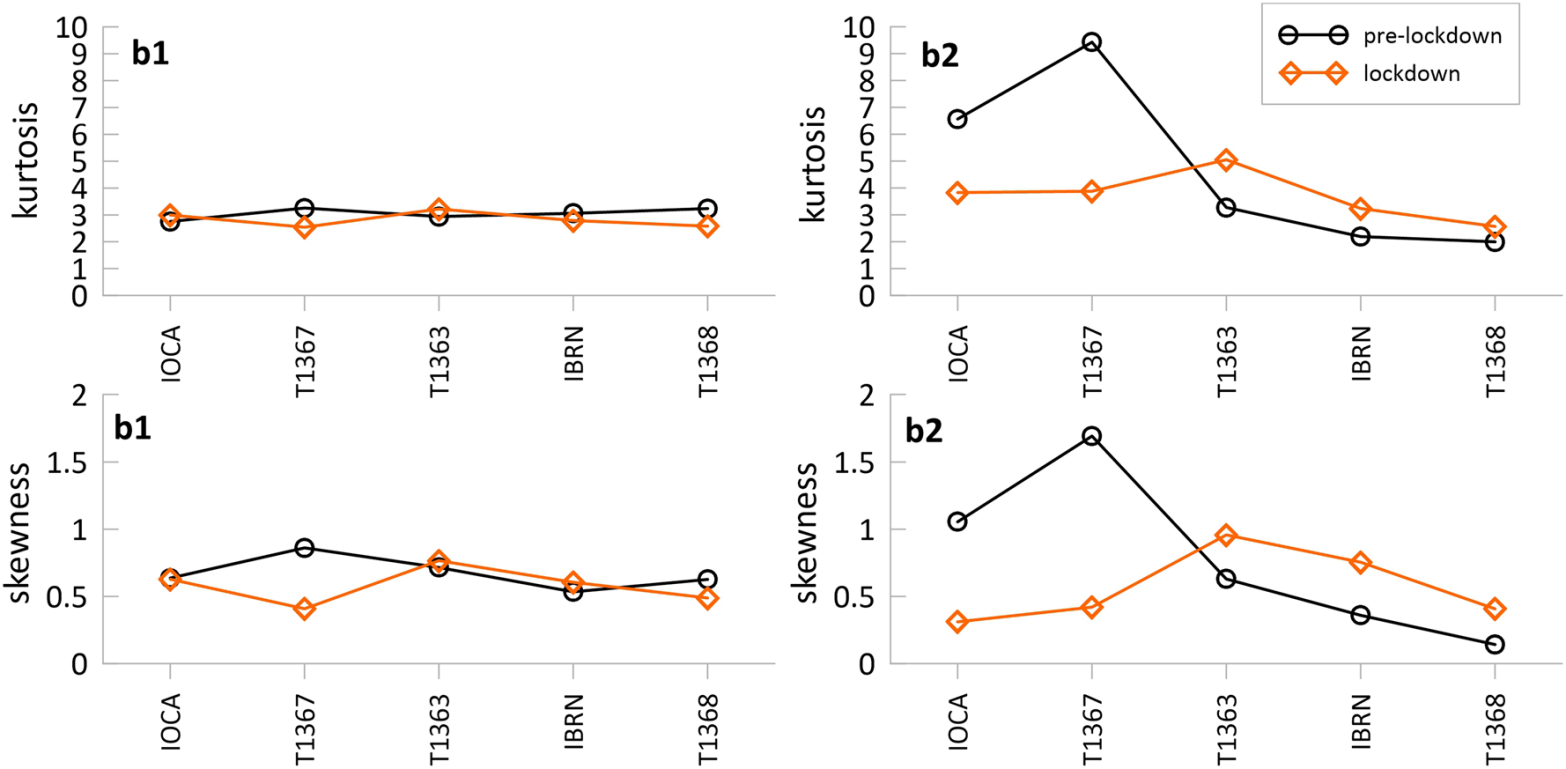
Kurtosis (upper panels) and skewness (lower panels) of the RMS distribution for each station calculated in b1 and b2, during the pre- (black line) and lockdown (orange line) periods.

As shown in Fig. 4, during the pre-lockdown period, the RMS distribution in b1 is essentially mesokurtic (k = 3) at all the stations, with a slight positive (< 1) skewness. During the lockdown, in both northern and southern stations, the distributions remain mesokurtic (or slightly platykurtic at T1367) and slightly skewed to the right. This suggests that the signal in b1 is not affected by the variations of the ambient conditions determined by the pandemic.

Instead, in b2, the RMS distributions at IOCA and T1367 are strongly leptokurtic and right-skewed during the pre-lockdown. Leptokurtic distributions of the seismic noise amplitude are related to the presence of short duration signals with large peak amplitudes, compared to the background^26^; in particular, the kurtosis of impulsive seismic signals related to the anthropogenic activity can take values far beyond five^27^. In such a framework, the marked high value of kurtosis at IOCA and T1367 stations (in pre-lockdown) could be attributed to the strong presence of human-induced activities. Kurtosis and skewness drastically decrease in lockdown and the distributions, although still leptokurtic (at a certain degree), tend to the gaussian shape. Slight variations of *k* and *s* are observed at T1363 comparing pre- and lockdown data.

The high pre-lockdown values of kurtosis and skewness, particularly evident at T1367 station, indicate a long-tailed right-skewed distribution ascribable to high-energetic transients, which overlap the low background signal (see the RMS levels Fig. 3) typical of the quiet site where T1367 is located (red zone).

To summarize, in b1, the northern stations show similar statistical moments in both pre- and lockdown periods, indicating a nearly Gaussian distribution. Hence, the silence of lockdown suggests that the anthropogenic noise does not affect b1.

In b2, at the northern stations the statistical moments change from pre- to lockdown and the RMS distributions deviate from supergaussianity towards a gaussian-like behaviour. In b2, during the lockdown, the kurtosis is always below five (tending to a Gaussian shape) suggesting fewer outliers, that might be related to fluctuations in energy of the source, since the anthropogenic noise is strongly reduced.

### Time decomposition: independent component analysis

By adopting Independent Component Analysis, which is based on fourth order statistical moments^19^, it is possible to check whether a time decomposition of the seismic noise is reliable in order to better constrain the features of the hydrothermal system. This can shed light on how the anthropogenic noise affects the basic source signal.

Indeed, ICA can provide information on the dynamical state of Ischia both in terms of complexity of the generation process (i.e. more modes of the same dynamical system) and different sources, if any, that are involved in the experimental series^29,30^.

**Figure 5:**
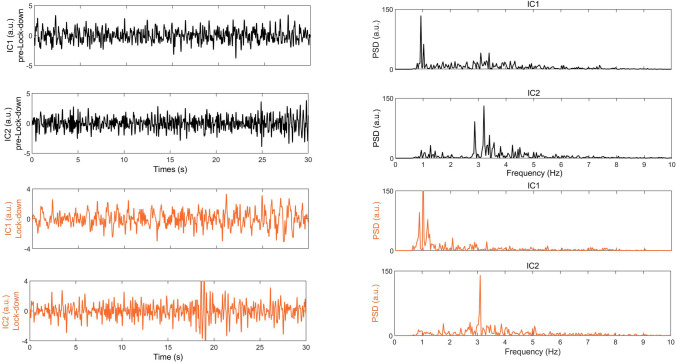
An example of ICA extraction: comparison between the decomposed signals (IC1 and IC2) in pre-lockdown (in black) and lockdown (in orange). As it can be seen, IC1 is always well peaked and extracted, whereas IC2 is much well extracted during the lockdown (a sharped frequency content vs a multiple-peak band). The original series in this example are relative to Jan 13, 2020, 15:32 UTC (in pre-lockdown) and Apr 09, 2020,15:32 UTC (in lockdown) considering all the stations.

The application of ICA, during pre- and lockdown, was carried out on segments of signals of 30 s extracted by using the same algorithm reported in Cusano et al.^13^ and band-pass filtered in 0.8–10 Hz frequency range^13^. We found two main independent signals, one peaked at 1.1 ± 0.2 Hz (IC1) and the second one at 3.2 ± 0.4 Hz (IC2). The results are reported in Fig. 5, where both the extractions in pre- and lockdown periods are shown. In analogy with what shown in Cusano et al.^13^, the black curve, which corresponds to IC1, was always extracted (and attributed to the permanent source), whereas IC2 was sometimes extracted but associated to multi-peaks in a wide range 2–4 Hz. As a consequence, no definitive indication was ascertained on its nature due to the contribution of the anthropogenic noise that insists on the same frequency band.

During the anthropogenic silence of the lockdown, IC2 is always extracted when the involved energy corresponds to certain values of RMS. Additionally, from a spatial point of view, when one considers as input of ICA only the signals recorded at the three stations on the northern area (IOCA, T1363, T1367) the performance of ICA improves. This is taken into account by the Principal Component Analysis^19^, that indicates an information content of at least 60% in that time period and, especially, for time windows which include the outliers (values of RMS above the threshold of the 90th percentile). This observation led us to perform a detailed analysis on time windows containing the outliers in the RMS estimated at IOCA and T1367 stations during the lockdown (see, Fig. 3). As a result, we find that IC2 is always extracted in presence of the outliers, that can emerge in lockdown, but that would be likely hidden in the noise in pre-lockdown.

This result is illuminating in indicating that both ICs are a source effect; indeed, the outliers underline physical conditions in which a higher energy is involved. In the absence of anthropogenic noise, we would not expect any IC2, once supposed it to be generated by human activity. In fact, IC2 does not depend on the day/night influence that, once again, excludes its anthropogenic nature. A further support to this consideration comes from the hourly distribution of the outliers in lockdown that does not show any cyclicity related to the anthropogenic activity (see, e.g., the period March 8–April 15 in Fig. 3).

Furthermore, avoiding the southern stations (T1368 and IBRN), the statistics in the extraction of IC2 strongly improves, suggesting that it is mainly concentrated and linked to the hydrothermal system in the North of the island and hence well detected by the northern stations.

Notice that the energy threshold value (Fig. 3) is hidden in the background noise in periods far from lockdown. Indeed, during pre- and post-lockdown phases, the two independent signals are always present but sometimes IC2 is not separated due to the strong presence of the anthropogenic noise that radiates energy in the same frequency band. In other words, these results clearly indicate that both ICs are source signals and are a fingerprint of the hydrothermal system in the North: IC1 is the dominant source; IC2 is its higher mode, activated when the energy is high enough.

## Discussion

In the present paper, we took the opportunity to study the seismic noise during the lockdown period. We concentrated our attention on Ischia Island, by performing a detailed investigation of the seismic noise in order to compare its characteristics between pre- and lockdown phases. Specifically, we analysed the spectral features, the energy release estimated by RMS, the statistical moments, and the decomposition of the wavefield in terms of independent components (ICs).

Summarizing, the presented results are:

1. The frequency content is always below 10 Hz, with 1–2 Hz band (b1) of greatest amplitude and the 2–4 Hz band (b2).
2. RMS evolution is basically the same in b1 both in pre- and lockdown, evidencing a stationary behaviour, whereas it experiences a strong amplitude drop in b2 due to the strong reduction of the anthropogenic noise. Nevertheless, the outliers underline time periods when a higher energy is involved.
3. The statistical moments evaluated on RMS distribution indicate that in b1 the northern stations have a similar distribution independently of the lockdown. In b2, instead, the RMS distributions tend to be Gaussian (slightly leptokurtic with kurtosis of about 4 and, thus, few outliers revealing fluctuations in energy of the source) from a supergaussian condition (more related to the human activity).
4. The background noise is decomposed into two independent modes, IC1 (1−2 Hz) and IC2 (2−4 Hz), both in pre- and lockdown, but with peculiar spectral signatures sharpened around 1 Hz and 3 Hz respectively in lockdown. IC1 is always the most energetic, while IC2 is always extracted in those time windows corresponding to an enhancement of the energy (outliers). This especially occurs restricting the analysis to the northern stations.

Putting together all previous results, a conceptual model can be derived. Starting from the seminal paper of Cusano et al.^13^, we demonstrate that the whispering at Ischia is basically due to two modes corresponding to IC1 and IC2, triggered by a transient pressure disturbance in the hydrothermal system located in the northern part of the island. IC1 is the fundamental mode and the most energetic; IC2 is the first higher mode activated as independent when a higher energy is involved in the source process. In pre-lockdown condition, this component was faint with respect to the background noise in the same frequency range. The lockdown represented the ambient conditions suitable to detect this mode due to the strong amplitude reduction of the anthropogenic noise.

The hydrothermal system acts like a fluid–solid structure which can produce a sustained signal like a whisper emitted by at least two almost identical organ pipes played side by side. Lord Rayleigh^31^ was the first to observe this phenomenon: “rather than each blaring their own tone, the two pipes will barely make a whisper. But put a barrier between them, and they sing loud and clear”. Apparently, the signal seems noisy, but it keeps some specific frequencies (the formant in the case of the speech, see e.g.^32^ and references therein) related to the dimensions of the source.

Following this parallel with the physics of the organ pipes^33–35^, as increasing the level of the involved energy, the system shows a nonlinear behaviour passing through different states: with one extracted mode (the fundamental), or with two extracted modes (fundamental and first mode) or even higher. The fundamental mode provides, in order of magnitude, the length of the vibrating solid structure.

Physical constraints on the source of excitation of the whisper can be derived integrating the present inferences with the outcomes of the recent hydrogeological, geochemical, and geophysical studies on Ischia Island. The hydrothermal system in the northern sector consists in a deep (> 900 m of depth) reservoir with equilibrium temperatures ~ 200–270 °C and a shallow aquifer (0–200 m of depth) mainly composed of both meteoric and sea water, mixed with the ascending deep hydrothermal fluids^15,17^. Moreover, magnetotelluric surveys show a low resistivity zone, presumably related to the shallow aquifer, located between the coast and the inner part of the island. This feature deepens to about 700–800 m down and has been ascribed to an interface between saline water and freshwater^36^. The upward fluid migration from the shallow aquifer produces surface manifestations such as fumaroles and hot springs. The fluid escape occurs along faults and fractures, which represent preferential paths^17,37^. The effect of the structural discontinuities on the fluid flow is particularly visible along the main faults bordering Mt. Epomeo and in the Casamicciola area. In fact, along these faults, fumarolic fields (Mt. Cito, Pizzone) and hot springs with large volumetric flow rate are located.

**Figure 6:**
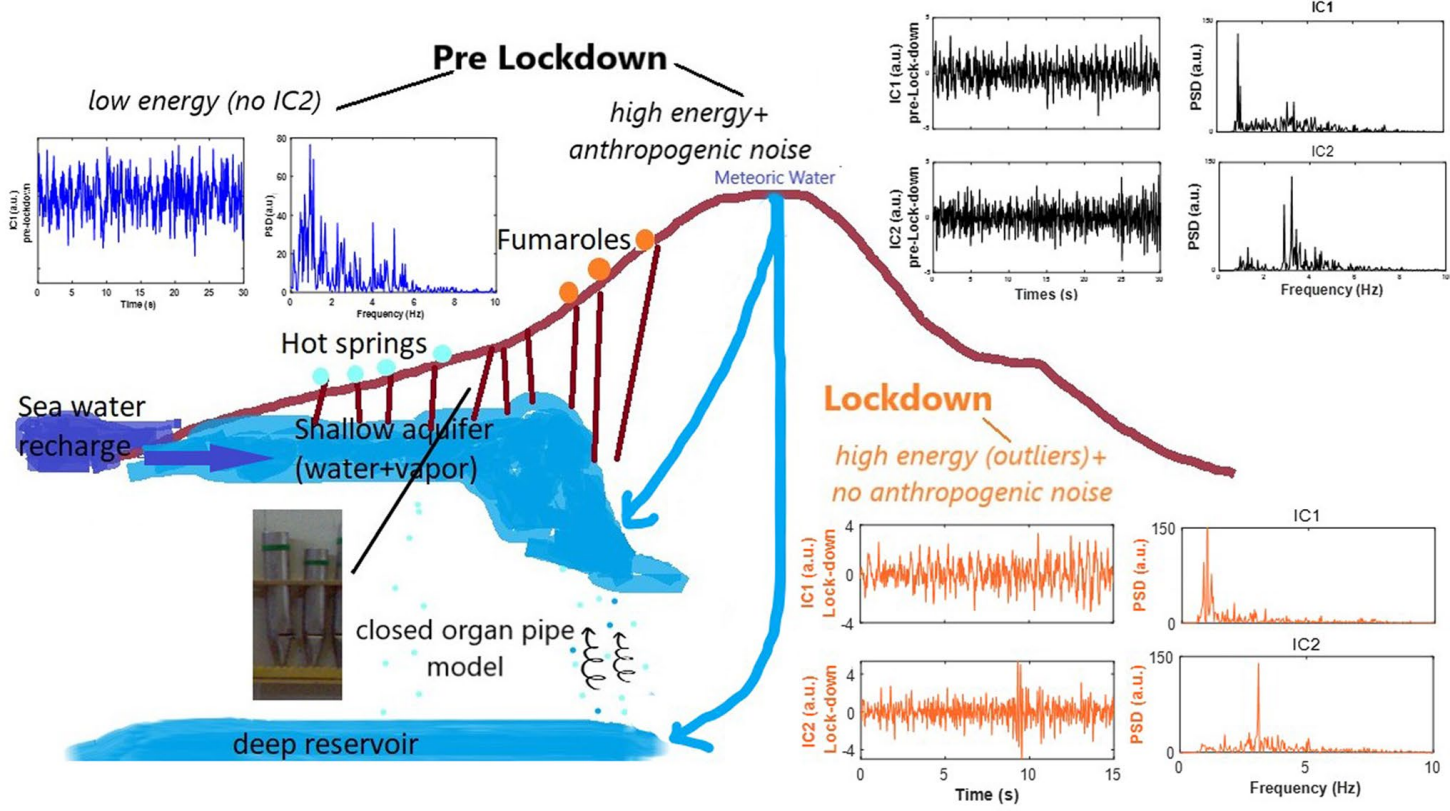
Sketch of the hydrothermal system at Ischia: functioning of the organ-pipe like model providing the whisper. The picture of the closed pipes is taken from the laboratory of Acoustics (Salerno University) and is representative of the behaviour of the system. The helicoidal arrows indicate fluid flow from the deep reservoir to the shallow aquifer, which in turn is mainly fed by meteoric and sea water; the red lines are representative of the faults, feeding fumaroles and hot springs. The panels show waveforms and spectra of the extracted components in pre-lockdown (blue and black lines), and lockdown (orange line).

Such geological framework suggests that we are dealing with a dendritic system of conduits plumbed by the shallow aquifer which acts as the wind-chest of an organ pipe, providing the whisper (Fig. 6). The excitation of higher energetic levels is likely triggered by the pressure build-up in the hydrothermal system, which can supply the additional energy required to activate the higher mode (IC2) of the pipe. This hypothesis is supported by the temporal variations of flux and temperature often observed at Mt. Cito fumaroles; the variability in the degassing pattern has been related to both pressure fluctuations and hydrothermal alterations occurring in the source fluids^18,38^. Such changes in a hydrothermal system can cause anomalous increases/decreases of groundwater temperature and fumarole flux flow^39–41^.

Evident variations in the fumarolic activity of Mt. Cito have also been reported in concomitance with local earthquakes^18,38^. In particular, that area is very close to the epicenter of the event occurred on 21 August 2017, whose origin has been attributed to a fault-valve type mechanism consistent with the periodic pressurization of the hydrothermal reservoir^37^. As shown by Cusano et al.^14^ the August 2017 earthquake and seismic noise share the same independent components (IC1, IC2), which are the characteristic modes of the pipes. Thus, according to our proposed model, the Ischia “organ-like system” can play both continuous whisper and transients, depending on the involved energy and how long the latter is sustained.

Different models have been introduced to take into account the source of volcanic/hydrothermal events in specific cases. Helmholtz resonator was applied to recover information about infrasound tornillos^42,43^ where a reservoir and a conduit could be identified.

Oscillations of bubble clouds have been also suggested to describe the solid-magma interaction in volcanic plumbing systems where some gas accumulates near localized asperities within the shallow magma chamber. As an example, this model was advocated as potential mechanism for volcanic tremor generation at Kilauea volcano, Hawaii^44,45^. In that mechanism, ascending bubbles of CO_2_ are expected to accumulate on geometrical irregularities on the walls of the shallow magma chamber.

Regarding more specifically the hydrothermal systems, some complex models, i.e. eddy shedding, slug flow and the soda bottle (see, e.g.^46^) have been advocated to explain the mechanism that sometimes generates the transition from a harmonic (in which, however, several overtones are involved) into a broadband tremor and vice versa.

Recent papers focus on the role of pipe-like structures in the dynamics of hydrothermal systems. For example, some geological structures at Yosemite National Park, California, show hydrothermal alteration concentrated along pipes, whose diameter is of the order of meters or more^47^**.** Moreover**,** in the Vøring Basin, offshore Norway, radial faults were associated with hydrothermal vents, represented by cylindrical- and conical-shaped conduits acting as pipes^48^.

For sake of clarity, in the case of Ischia Island, we are dealing with a hydrothermal tremor generated by a geothermal system made of a network of conduits. In general, the tremor source process can be characterized by a mean length scale that may correspond, for example, to a conduit filled with fluid^49^. In line with these thoughts, the collective behaviour of such a complex system can be ideally assimilated to an equivalent pipe, whose length can be estimated, providing indications both on the spatial scales involved in the phenomenon and on the physical behaviour of the system (i.e. the appearance of more modes in some ratios).

Unfortunately, the nonlinear model able to fully take into account the observed self-oscillations produced by a vibrating pipe is not available yet. Anyway, the linear approximation can provide reliable estimates of the geometric parameters of the equivalent pipe. Indeed, such parameters, in the case of a pipe with large width, can be estimated including the open-end correction.

Having evidenced two activated modes at Ischia, i.e. the fundamental and the first odd harmonics, we suppose to deal with a closed cylindrical pipe, which can excite only odd harmonics^33–35^. For a closed pipe with radius *r*, the following equations hold^50–52^:

1 $${L}_{eff}=L+\Delta L=L+\alpha r=\frac{c}{4f}$$

2 $$Q=\frac{4L{L}_{eff}}{\pi {r}^{2}}$$

3 $$r=\frac{c}{4f}\frac{1}{\left(\sqrt{\frac{{\alpha }^{2}}{4}+\frac{\pi Q}{4}}+\frac{\alpha }{2}\right)},$$

where *L*_*eff*_ is the effective pipe length; *L* is the equivalent pipe length; $\Delta L$ is the open-end correction; $\alpha$ is about 0.8^51^; c is the sound speed; *f* is the frequency of the fundamental mode; $Q$ is the quality factor of the pipe. Since $Q=$ $\frac{f}{\Delta f}$, in our case it is equal to about 30; this value was experimentally estimated by evaluating the width ($\Delta f)$ of the spectrum of IC1.

In a water–vapor mixture, the sound speed of the two-phase fluid as a function of pressure (*P*) and temperature (*T*) is^53^:

4 $$c=\left({x}_{m}{\left(\frac{{g}_{\nu }}{P}\right)}^{\frac{1}{\gamma }}+\frac{1}{{\rho }_{l}}\right){\left(\left(1+{x}_{m}\right)\left(\frac{{x}_{m}{g}_{\nu }^{\frac{1}{\gamma }}}{\gamma {P}^{\frac{1+\gamma }{\gamma }}}+\frac{1}{{\rho }_{l}{K}_{l}}\right)\right)}^{-\frac{1}{2}}$$

where ${x}_{m}$= $\frac{{M}_{\nu }}{{M}_{l}}$ is the vapor–liquid mass fraction; ${g}_{\upnu }=\frac{TR}{M{\rho }_{\upnu }^{\gamma -1}}$, with ρ_ν_ and ρ_*l*_ being the vapor and pure liquid densities, respectively, *R* = 8.32 J K^−1^ mol^−1^ is the universal gas constant, *M* = 18.02 g mol^−1^ is the water molecular weight, and γ = 1.31 is the isentropic exponent of steam; ${K}_{l}$ is the bulk modulus of the pure liquid phase.

Sound speed curves as a function of pressure, temperature and vapor mass fraction have been derived from Eq. (4) by a number of authors^53,54^. By assuming pressure and temperature in the range [1–50] bar and [100–264] °C, respectively, which are reasonable values for the Ischia aquifer up to 500 m depth, the sound speed *c* spans over the range [1400–1000] m/s, for low (10^–7^–10^–6^) vapor mass fraction.

Thus, considering the above equations, we estimate an equivalent pipe length between [300–200] m and radius between [70–50] m for the mode at 1 Hz. The equivalent pipe provides the characteristic mean scale length on which the phenomenon occurs, and the estimated range of its dimension is compatible with the size of the system of faults in the North of Ischia^55^.

This conceptual model clears up the mechanism of generating both seismic noise (whisper) and the transients^14^ linking them to a common phenomenon of fluid–solid interaction in the hydrothermal system of Ischia Island. More than that, our experimental results put physical constraints for modelling the source mechanism once the medium properties, such as variation in fumaroles flux and/or hydrothermal alteration, are considered. Specifically, a mean field approximation model, based on nonlinear equations, as in the case of the self-sustained musical instruments, could recover some aspects of the observed phenomena.

A crucial element of the model should be represented by a time-dependent amplitude threshold, which takes into account the variability of the deep and superficial recharges and the bubble flux.

Finally, the observations, shown in the present paper, open the route to forecasting: indeed, an amplitude signal (detected as RMS outliers) overcoming a certain threshold can track a departure of the system from the equilibrium state (seismic noise) to the non-equilibrium (transients, earthquakes).

## Methods

In this section, we provide details on the data that we used in the present paper and on the Independent Component Analysis technique.

### Seismic data acquisition

The seismic network of Ischia Island is managed by Istituto Nazionale di Geofisica e Vulcanologia (INGV) and is composed of permanent^22^ and temporary^23^ stations. On August 27, 2017 a M_D_ 4 earthquake and the following seismic sequence struck the island and caused two victims and severely damaged the buildings inside the town of Casamicciola (Fig. 1). Consequently, the previous monitoring network, which comprised IOCA station (Fig. 1), was improved with other permanent and mobile stations. For the present study, we used the stations operating in the northern (IOCA, T1363 and T1367) and southern (IBRN and T1368) sectors of the island in the period January 1, 2020–June 19, 2020. The stations are equipped with the following sensors: LE3D5s (T1363), 1 Hz LE3Dlite (T1367), LE3D20s (T1368), and Guralp CMG − 40 T (IOCA and IBRN). The acquired signals are telemetered in near-real-time to the monitoring center in Naples (INGV- Osservatorio Vesuviano). IOCA is taken as a reference station as it operates the longest in the areas of interest. The dataset used in this study consists of continuous recordings.

### Independent component analysis

Independent Component Analysis (ICA) is an entropy-based technique, able to recognize underlying sources from multivariate statistical data^19^. It exploits the statistical independence of the sources, by using fourth-order statistics. Seismic data are supposed to be a linear representation of non-Gaussian sources and the extracted components are as independent as possible. The Central Limit Theorem is the mathematical basis underlying the method: “given two independent random variables, the distribution of their sum is close to a Gaussian, more than the distribution of either of the variables”. ICA has been already successfully applied to a variety of experimental signals such as in the field of volcano seismology and oceanography^20,29,30,56,57^. Briefly, ICA model assumes to have m recordings **x** that are hypothesized to be the linear superposition of n mutually independent unknown sources **s**. The superposition is taken into account in a matrix (**A**) essentially due to path, noise, instrumental transfer-functions, etc. Formally, the mixing model is written as **x** = **As**. Under these hypotheses, ICA provides a separating matrix **W** = **A**^−1^, in such a way that the vector **IC** = **Wx** is an estimate **IC** ∼ **s** of the original independent sources. In practice, computationally, we use the fixed-point FastICA algorithm^19^.

## Data Availability

All relevant data are available from the authors.
